# Mitochondrial Genome Sequence of the Scabies Mite Provides Insight into the Genetic Diversity of Individual Scabies Infections

**DOI:** 10.1371/journal.pntd.0004384

**Published:** 2016-02-12

**Authors:** Ehtesham Mofiz, Torsten Seemann, Melanie Bahlo, Deborah Holt, Bart J. Currie, Katja Fischer, Anthony T. Papenfuss

**Affiliations:** 1 Bioinformatics Division, The Walter and Eliza Hall Institute of Medical Research, Parkville, Victoria, Australia; 2 Department of Medical Biology, University of Melbourne, Melbourne, Victoria, Australia; 3 Victorian Life Sciences Computation Initiative, University of Melbourne, Melbourne, Victoria, Australia; 4 Department of Mathematics and Statistics, University of Melbourne, Victoria, Australia; 5 Menzies School of Health Research, Charles Darwin University, Casuarina, Northern Territory, Australia; 6 QIMR Berghofer Medical Research, Herston, Queensland, Australia; 7 Sir Peter MacCallum Department of Oncology, University of Melbourne, Melbourne, Victoria, Australia; 8 Peter MacCallum Cancer Centre, East Melbourne, Australia; University of California San Diego School of Medicine, UNITED STATES

## Abstract

The scabies mite, *Sarcoptes scabiei*, is an obligate parasite of the skin that infects humans and other animal species, causing scabies, a contagious disease characterized by extreme itching. Scabies infections are a major health problem, particularly in remote Indigenous communities in Australia, where co-infection of epidermal scabies lesions by Group A Streptococci or *Staphylococcus aureus* is thought to be responsible for the high rate of rheumatic heart disease and chronic kidney disease. We collected and separately sequenced mite DNA from several pools of thousands of whole mites from a porcine model of scabies (*S*. *scabiei* var. *suis*) and two human patients (*S*. *scabiei* var. *hominis*) living in different regions of northern Australia. Our sequencing samples the mite and its metagenome, including the mite gut flora and the wound micro-environment. Here, we describe the mitochondrial genome of the scabies mite. We developed a new *de novo* assembly pipeline based on a bait-and-reassemble strategy, which produced a 14 kilobase mitochondrial genome sequence assembly. We also annotated 35 genes and have compared these to other Acari mites. We identified single nucleotide polymorphisms (SNPs) and used these to infer the presence of six haplogroups in our samples, Remarkably, these fall into two closely-related clades with one clade including both human and pig varieties. This supports earlier findings that only limited genetic differences may separate some human and animal varieties, and raises the possibility of cross-host infections. Finally, we used these mitochondrial haplotypes to show that the genetic diversity of individual infections is typically small with 1–3 distinct haplotypes per infestation.

## Author Summary

The scabies mite is a skin parasite that infects humans and other animal species, causing scabies, a contagious disease characterized by extreme itching. Scabies infections are a major health problem in developing countries and in indigenous Australian populations, where scabies is associated with pyoderma (skin sores) and linked to rheumatic fever and rheumatic heart disease. Little is known about the genetics of the scabies mite. We have assembled the mitochondrial genome of scabies mites obtained from human patients in Australia and from a pig model. While investigating the genetic diversity of each infestation, we found that mitochrondial genomes clustered into two broad clades and showed limited genetic diversity within each infestation. Remarkably, one closely related clade included both human and pig mites, suggesting that mite transmission from pig to human may be possible. This could have major implications in the management of porcine mange and human scabies.

## Introduction

The scabies mite is an ectoparasitic arachnid that causes an itchy skin infection, known as scabies. Each year around 300 million people worldwide are affected by scabies [1]. Scabies is responsible for a significant disease burden in affected populations through its obligate parasitic lifecycle, which facilitates secondary infections by other pathogens. A severe, but more rare form of scabies, known as crusted scabies, is characterised by hyper-infestation. It generally occurs in immune-compromised individuals [2], although it can occur in patients with no overt immunological deficiency [3]. Cases of crusted scabies can play a significant role in transmission [4]. The mite also infects more than a hundred species of mammals, creating an animal welfare and economic burden in primary industry [5–7].

Scabies represents a major health problem in many remote Indigenous communities in Australia and particularly affects children. Up to 25% of adults and 50% of children acquire scabies infections each year, and 7 out of 10 children under 1 year contract scabies with first presentation peaking at 2 months of age [4, 8]. Scabies is associated with pyoderma (skin sores) in Indigenous communities, but also in many other circumstances of disadvantage globally [9]. In tropical regions, the major pathogens of pyoderma are Group A streptococcus (*Streptococcus pyogenes*; GAS) and *Staphylococcus aureus;* with *S*. *pyogenes* considered the dominant and usually primary pathogen [4, 10, 11]. This is also the case for remote Indigenous communities in northern and central Australia [4, 10–12]. Sequelae of infection with GAS include acute post-streptococcal glomerulonephritis, which can be clustered (epidemic) or sporadic, and acute rheumatic fever [4, 13, 14]. Rheumatic heart disease, characterised by heart valve damage, occurs as a consequence of acute rheumatic fever and repeated episodes of it can result in cumulative heart valve damage, with consequent heart failure and death [5, 13]. The prevalence of rheumatic heart disease in Indigenous communities is amongst the highest in the world [4, 15]. Several recent studies provide molecular evidence of the scabies mite itself promoting streptococcal growth in pyoderma through complement inhibitors [1, 16, 17]. Association of scabies with these long-term health problems makes it an important factor to consider in Indigenous health. Despite this, there is a relative paucity of genetic and genomic information on scabies [1].

The scabies mite belongs to the subclass Acari (Arthropoda: Chelicerata: Arachnida: Acari), which contains around 48,000 species of ticks and mites [18], and order Sarcoptiformes. Currently, the scabies mite is classified taxonomically as a single species with different varieties based on host specificity [19]. Reportedly, cross infectivity is rare and when it happens it is generally of temporary nature and self-limiting infestation [19, 20]. However, evidence regarding host specificity is conflicting, with some studies suggesting cross infectivity is possible between certain animal and human varieties [21, 22], while others suggest mono-specificity of scabies mite varieties [4, 5, 23–26]. For example, genetic and phylogenetic studies that used hypervariable satellite markers, 16S rRNA and cytochrome oxidase subunit I of mitochondria (cox1), have shown that human and dog mites are genetically distinct in north Australian sympatric populations and that gene flow between the scabies mite population is extremely rare [19, 27]; while another study, using the cox1 gene showed that dog mites from China, USA and Australia are genetically similar to with certain human mites from Australia [21, 22]. Choice of the genetic marker may play a role in conflicting outcomes. Additionally, it has been postulated that each variety, when infesting a non-native host, can acquire morphological and innate characteristics suited to the host through selection if it is allowed to persist due to immunodeficiency or malnutrition of the host [20].

Host specificity has been an area of ongoing investigation because potential cross infectivity, in particular between domestic and companion animals and humans, would have important implications for disease control programs. There are no morphological differences between mites from different host species [20], but multiple failed experimental cross infestation attempts indicated that physiological differences may exist [1, 27, 28]. Mites from dogs have successfully established long term infestations in rabbits [28]. Microsatellite studies in wild animal mite populations also indicated a limited gene flow between mites from sympatric host populations [29], but a further study suggested a prey to predator transfer of mites may be possible [30]. Taken together, available data suggests that a limited gene flow occurs between host-associated populations of scabies mites, however a strict ‘host taxon’ law cannot be assumed.

Here, we describe the *in silico* isolation, *de novo* assembly and analysis of the mitochondrial genome of scabies mites from a variety of complex metagenomic samples. The samples consisted of thousands of whole mites collected from two clinical isolates from different regions of Australia and replicates from a laboratory porcine model of scabies [31]. We assembled the mitochondrial genome by applying a bespoke, iterative bait-and-assemble strategy to the massively parallel sequencing data from each mixture—demonstrating the utility of the general approach on complex metagenomics mixtures. Additionally, we identified 6 mitochondrial haplotype clusters in the human (*Sarcoptes scabiei* var. *hominis*) and pig (*Sarcoptes scabiei* var. *suis*) scabies/mange mite populations sampled. This allowed us to examine the genetic diversity within and between isolates and suggests an extremely low level of diversity overall. To the best of our knowledge, these findings provide the first view of the genetic diversity of individual scabies infestations (intra-host diversity) based on whole mitochondrial genome sequences.

## Methods

### Collection and DNA extraction

The collection of human patient samples was approved by the Human Research Ethics Committee of the Northern Territory Department of Health and Menzies School of Health Research (approval 13–2027) and informed consent was obtained in writing from each participant. Animal care and handling procedures used in this study followed the Animal Care and Protection Act, in compliance with the Australian code of practice for the care and use of animals for scientific purposes, outlined by the Australian National Health and Medical Research Council. The study was approved by the QASP and the QIMR Berghofer MRI Animal Ethics Committees (DEEDIAEC SA2012/02/381, QIMR A0306-621M).

Skin scrapings were collected from two unrelated patients with severe crusted scabies (patient A and patient B) from different regional areas of Northern Territory, Australia. The collections from individual patients were made 14 months apart and were likely independent. On both occasions, scabies mites (*S*. *scabiei* var. *hominis*) were individually picked from the skin. Each sample contained >1000 mites. Two samples of pig mites (*S*. *scabiei* var. *suis*) were collected from an inbred population of mites from a pig model [31]. The samples were taken from different pigs from consecutive cohorts (where infections are passed on to new piglets from a previous group) at different time points. The first sample consisted of >1000 whole adult mites (pig unwashed). The second sample also consisted of >1000 mites, but was split into three subsamples, which were washed using different protocols to reduce the amount of bacteria present on the surface of the mites due to the wound micro-environment (pig washed 1, 2 and 3 respectively). The protocol entailed: (1) 15 min wash at room temperature in 4% paraformaldehyde in water [32]; (2) 1 hour incubation at 37°C in 150 mM NaCl, 10 mM EDTA, pH8.0, 0.6% SDS, and 0.125 ug/ul lysozyme [adapted from 33] and; (3) 1 hour incubation at 37°C in 1% bleach (Sodium hypochlorite) in water. Mites were subsequently rinsed twice in water. Between wash steps mites were centrifuged at 10000 rpm for 2 min.

### Whole genome sequencing

Whole mites were crushed and DNA was extracted from each sample using a Blood and Cell culture DNA Kit QIAGEN and a modified procedure, adapted from the manufacturer’s protocol. Washed mites were submerged in 1 ml of ice-cold lysis buffer (20 mM EDTA, 100 mM NaCL, 1% TritonX-100, 500 mM Guanidine-HCl, 10 mM Tris pH7.9) and homogenized with stainless steel beads of 2.8mm diameter at 6800rpm, 3 cycles, 30 sec per cycle, 30 sec between cycles. The suspension of lysed mites was supplemented with DNase free RNase A to 0.2 mg/ml and with Proteinase K to 0.8 mg/ml and incubated at 50°C for 1.5 h. After centrifugation at 4000g for 10 min to pellet insoluble debris the genomic DNA was isolated on the QIAGEN genomic tip as instructed in the manufacturer’s protocol. Six DNA libraries were constructed and 100 nucleotide (nt) long paired-end reads were generated on an Illumina HiSeq 2500. Additionally, 54 *S*. *scabiei* var. *suis* eggs were collected from the pig model. To reduce any surface bacteria, these were washed washed twice in 4% paraformaldyde and rinsed twice in water. DNA was extracted separately from 51 individual eggs, and from 3 pools of 5 eggs and 1 pool of 16 eggs. Sequencing libraries were constructed using the Nugen Ovation SP Ultralow DNA kit for 3 single eggs, and pools of 5 and 16 eggs, and sequenced using an Illumina HiSeq 2500 with 2x100 nt reads. Raw data is available via ENA accession PRJEB12428.

### Initial *de novo* assembly

Sequence read quality was assessed using FASTQC [34]. Adapter and quality (Q≥20) trimming was performed using TrimGalore! (v0.3.1) [35]. For the human and unwashed pig samples, reads were *de novo* assembled using Velvet (v1.2.08) [36]. To establish the best k-mer size, we initially assembled all reads from patient B using k-mer values of k = 61, 63, 65, 67, 69, 71, 73, 75, 79, 85, 89 and 95. Based on the quality of these assemblies, we used k = 69, 75, 77, 79, 81, 83, 85, 89 and 95 for patient A and the unwashed pig samples.

### Metagenomic profiling

Metagenomic profiling of the patient B mite assembly was carried out using PhymmBL (v4.0) [37]. We augmented the bundled PhymmBL model database with interpolated Markov models trained on the spider mite, *Tetranychus urticae* (strain London) (available at https://bioinformatics.psb.ugent.be/gdb/tetranychus/) [38] as a proxy for scabies mite. To estimate the species abundance, reads were aligned back to contigs using Bowtie2 (v2.2.3) in local alignment mode [39] and the number of reads aligning to each contig were counted.

### *De novo* assembly of the mitochondrial genome

**Fig 1:**
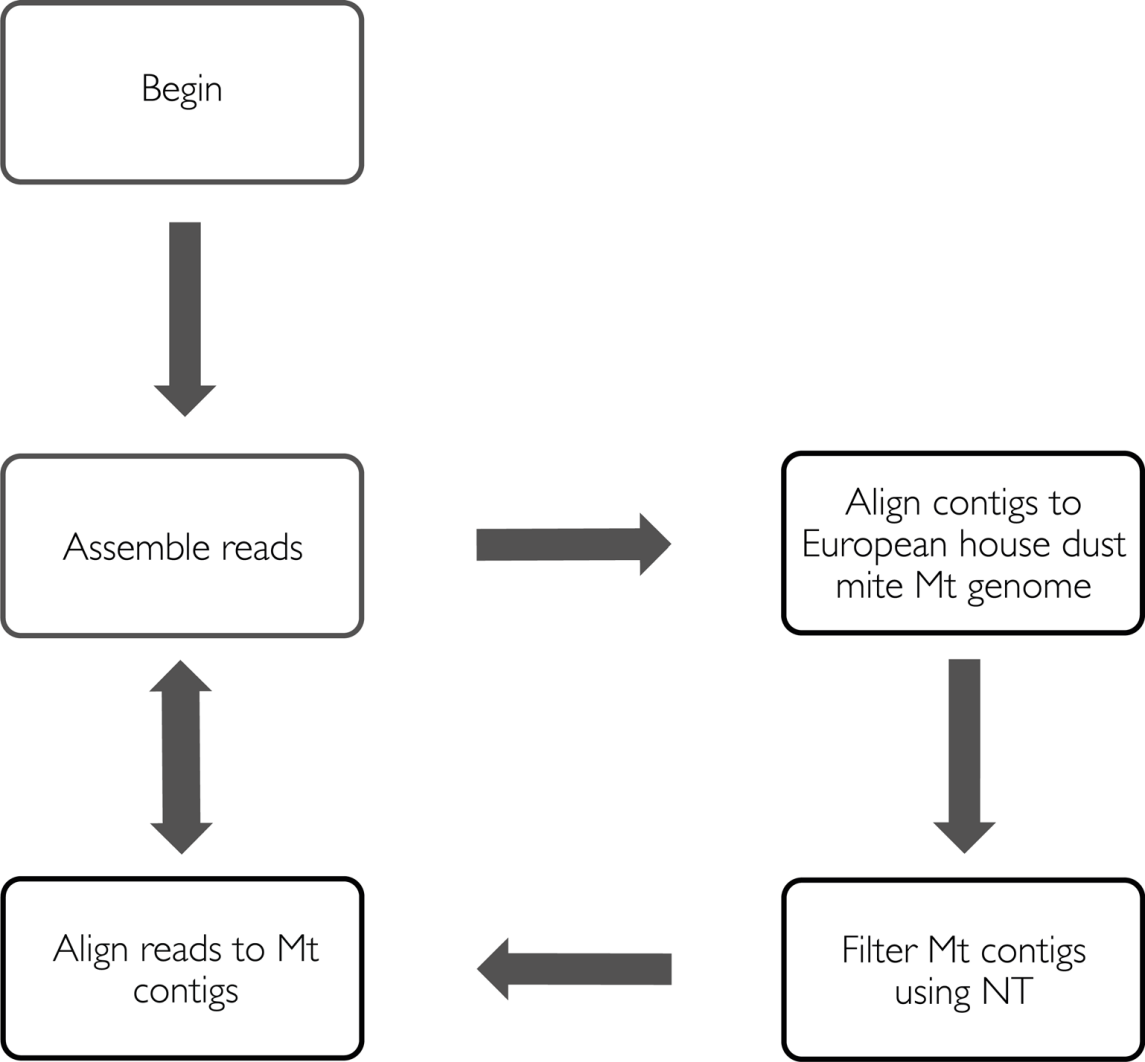
Bait-and-assembly workflow for metagenomics data. Two iterations were performed. NT stands for nucleotide collection blast database (nt) from NCBI.

For the two human samples and the unwashed pig sample, the mitochondrial genomes were assembled individually using a bait-and-reassemble strategy (see Fig 1 for overview). First, contigs from the whole genome assemblies were aligned to European house dust mite (EHDM), *Dermatophagoides pteronyssinus* mitochondrial reference genome (NCBI GenBank: EU884425) [40] using LASTZ (v1.02.00) [41] with default settings. For patient B, contigs from the k = 65 assembly were used for alignment; for patient A, contigs from k = 77, 79 and 89 were used; and for the unwashed pig sample, contigs from the k = 69 and 81 assemblies were used. These different k-mer value assemblies varied in their N50 values, largest contigs sizes and median read coverage. The aligned contigs were then filtered against the National Center for Biotechnology Information (NCBI) NT database for scabies mite mitochondrial contigs using BLASTN (v2.2.29) [42] to remove likely false positives due to homology with other mitochondrial genomes (e.g. host mitochondrial genome). After the filtering step, contigs from patient B k = 65 assembly gave the best coverage of EHDM mitochondrial reference genome. Adapter and quality trimmed reads from the patient B sample were then aligned back to the filtered patient B k = 65 contigs using Bowtie2 (v2.2.3) in local alignment mode. Aligned reads were then used to *de novo* assemble the patient B scabies mite mitochondrial genome using Velvet (v1.2.08). Assemblies were run using k = 69, 79, 87, 89, 91 and 95. A k-mer size of 91 gave the longest single contig and was selected as the reference mitochondrial genome of scabies mite.

To generate the mitochondrial reference for patient A, adapter and quality trimmed reads from the patient A sample were aligned to the patient B mitochondrial reference genome, and matching reads were *de novo* assembled using Velvet. The final contigs were scaffolded and gaps filled with Ns. This scaffold was then used to realign the patient A reads and reassemble. This process was run iteratively. The second iteration gave the minimal number of Ns in the contig and this was retained as the patient A mite mitochondrial genome.

The same process was used for the unwashed pig sample, but in this case the first iteration gave the best contig with minimal Ns.

### Annotation

Mitochondrial protein-coding genes were predicted on patient B mitochondrial reference genome using the MITOS pipeline (accessed online, August 2014) [43] using the invertebrate mitochondrial genetic code (with default settings). To verify the annotations, open reading frames (ORFs) between stop to stop codons were extracted from the assembled patient B mite genome using the GETORF program from the EMBOSS package (v6.6.0) [44] using the invertebrate mitochondrial codon table. The extracted 774 ORFs were then used to search the NCBI NR protein database using BLASTP (v2.2.29) (E-value threshold = 0.1). For further verification, 13 protein-coding genes from EHDM were aligned to the patient B mite reference genome using TBLASTN (v2.2.29).

12S and 16S ribosomal RNA were annotated using covariance models for those genes using Infernal (v1.1rc4) [45, 46]. The covariance models were built from multiple sequence alignments of 12S and 16S rRNA genes from three sarcoptiformes, *Steganacarus magnus* (NCBI RefSeq: NC_011574), *Dermatophagoides pteronyssinus* (NCBI GenBank: EU884425) and *Dermatophagoides farinae* (NCBI RefSeq: NC_013184) using Clustal-omega (run on the web version on 15/12/2013) [47]. MITFI (within the MITOS pipeline) was used to identify 21 of 22 standard mitochondrial tRNA genes.

### Single nucleotide polymorphisms and diversity

For each of the six samples, reads from each sample were aligned to the patient B reference genome using the Bowtie2 aligner in local mode. Pileups were generated using SAMtools (v0.1.19-44428cd) [48] mpileup. Varscan (v2.3.6) [49] was then used to call SNPs with the mpileup2snp command (Min Coverage: 300; Min Variant Frequency: 0.01; otherwise default parameters). SNPs called within the 100 nt boundary at both ends of the genome (positions 1–100 and 13,820–13,919) were manually filtered out due to lack of reliability of alignments at those low complexity regions. For each sample, SNP frequency was estimated using the ratio of reads supporting the variant to total read depth at the SNP.

## Results

Sequencing of the two human and four pig samples (unwashed and 3 washed technical replicates) generated 46 (patient B) to 62 (pig washed 1) million read pairs per sample (S1 Table). As expected, the libraries also contained host and microbial DNA. We estimated the level of contamination using two approaches. PhymmBL was applied to the patient B mite mitochondrial assembly to classify contigs into taxa. This revealed that 44% of contigs were from bacteria or other non-arachnid species (S1A Fig). Species abundance was estimated by re-aligning the reads back to the contigs. This revealed that 6% of the reads were derived from contaminants (S1B Fig).

Using our bait-and-assemble strategy, we assembled the mitochondrial genome of each sample (ENA accessions: LN874268-LN874270). The patient B mitochondrial genome assembly comprised a single contig of length 13,919 nt. The patient A and unwashed pig assemblies were 13,902 and 14,044 nt respectively. Both also consisted of single contigs, but each contained a contiguous gap of 118 (patient A) to 255 (unwashed pig) Ns. We were unable to generate a circular mitochondrial genome sequence and found the contig ends were composed of repeat rich sequences. Assuming the scabies mitochondrial genome is circular, we estimate that an AT-rich gap of approximately 300 nt in length is present in the human mite reference assembly. Similar gaps are also present in the other assemblies.

Similar to other sequenced Acari mitochondrial genomes, the scabies mite mitochondrial genome is also highly AT-rich with a 19.32% GC-content. The strand bias is characterized by GC and AT skews, calculated using (G%-C%)/(G%+C%) and (A%-T%)/(A%+T%) respectively. The GC skew for the leading strand is -0.0327 and AT skew is 0.0341. Negative GC skew and positive AT skew are similar to standard metazoan genome strand biases including Acari. Reversal in GC skew in terms of strands has been observed in only two mites so far, *Varroa destructor* [40, 50] and house dust mite [40].

### Annotation

**Fig 2:**
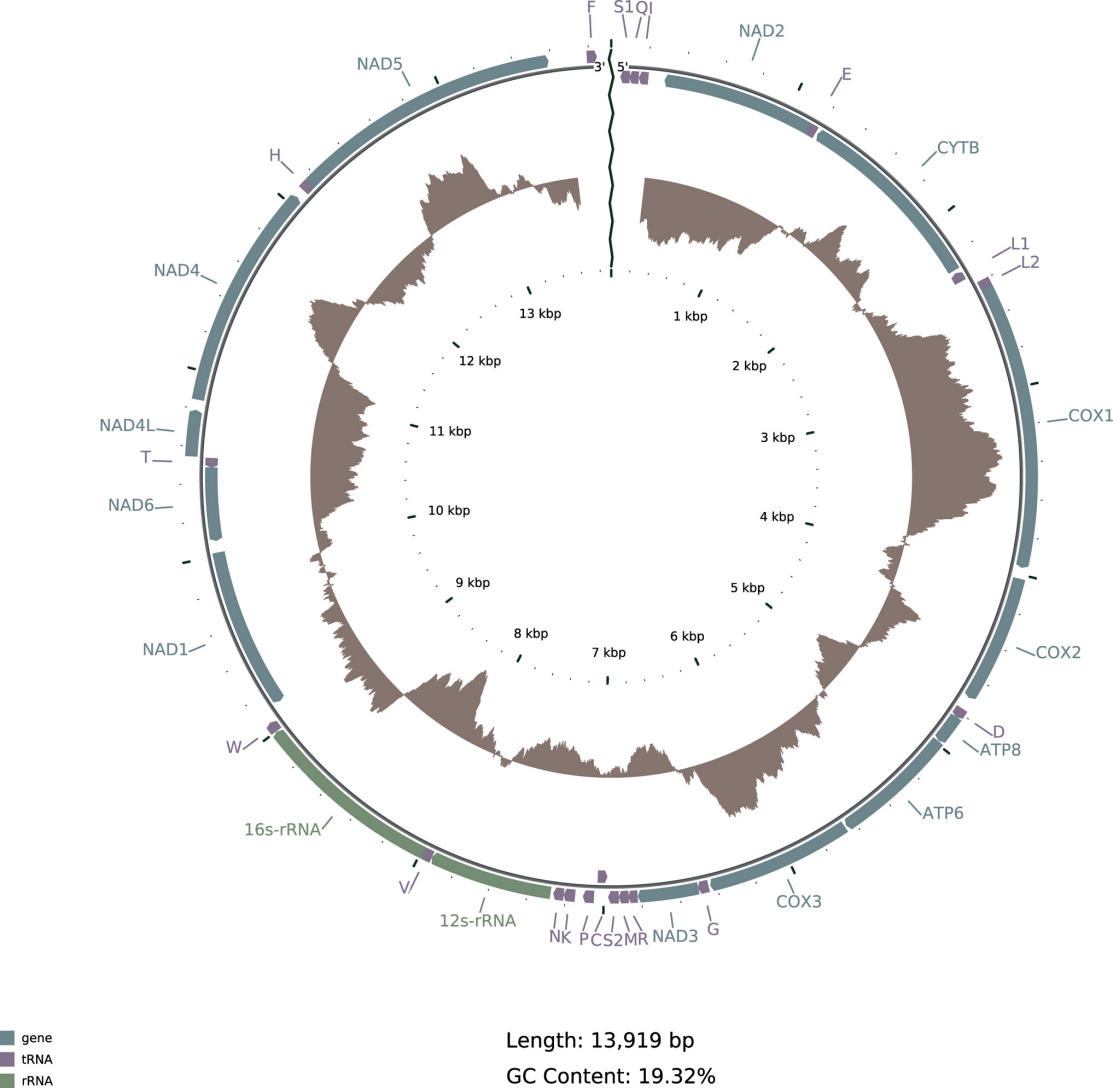
Annotation of the scabies mite, *Sarcoptes scabiei (*var. *hominis)*, mitochondrial genome. tRNA genes are abbreviated using the one letter amino acid code. The inner brown plot is plotted for GC content using window size of 500 and step 1. The plot shows deviations from the average GC content of the genome (0.1932). The plot is scaled based on the maximum (0.3034) and minimum (0.1078) GC content values.

Thirty-five predicted genes were identified in the scabies mite (var. *hominis*) mitochondrial genome (Fig 2). These include 13 protein coding genes, two ribosomal RNA genes, and 20 tRNA genes. All of the expected mitochondrial genes for a typical metazoan mitochondrial genome were identified, except for two tRNAs (Alanine and Tyrosine). Search with BLASTP of extracted stop-to-stop ORFs from the mitochondrial genome also verified 12 of the 13 protein-coding genes (except the ATP8 gene) and TBLASTN alignment of 13 protein coding genes from the EHDM mitochondrial genome to the scabies mite mitochondrial genome also confirmed 12 of the 13 protein coding genes (except the ND4L gene) in the scabies mite mitochondrial genome. Two tRNA genes identified by tRNAscan-SE were considered ambiguous due to their overlap with other genes (A is overlapping with C and Y is overlapping with NAD4L). However, the overlap is not on the same strand.

The gene order and strand specification of the protein coding and ribosomal RNA genes are the same as that of EHDM. All identified tRNA genes, except tRNA-C and tRNA-V also maintain the same gene order and strand as EHDM. The tRNA genes that are not syntenic with EHDM (tRNA-C, tRNA-V) are also on the opposite strand to that of EHDM.

### Single nucleotide polymorphisms and mitochondrial haplotypes

To identify genetic polymorphisms in the sequenced mite pools, reads were mapped back to the reference Mt genome (patient B). The average depth of coverage was 2914 across samples (average per sample ranging 1698–4299). A total of 665 single nucleotide polymorphisms (SNPs) were identified across all samples relative to the reference Mt genome (patient B assembly): 601 SNPs in the patient A sample, 598 SNPs in the patient B sample, and 102 SNPs in the unwashed pig sample, while the washed pig samples (w1, w2, w3) contained 102, 100, 102 SNPs respectively (S2 Table). The four pig mite samples are effectively replicates (biological and technical) and were highly concordant.

**Fig 3:**
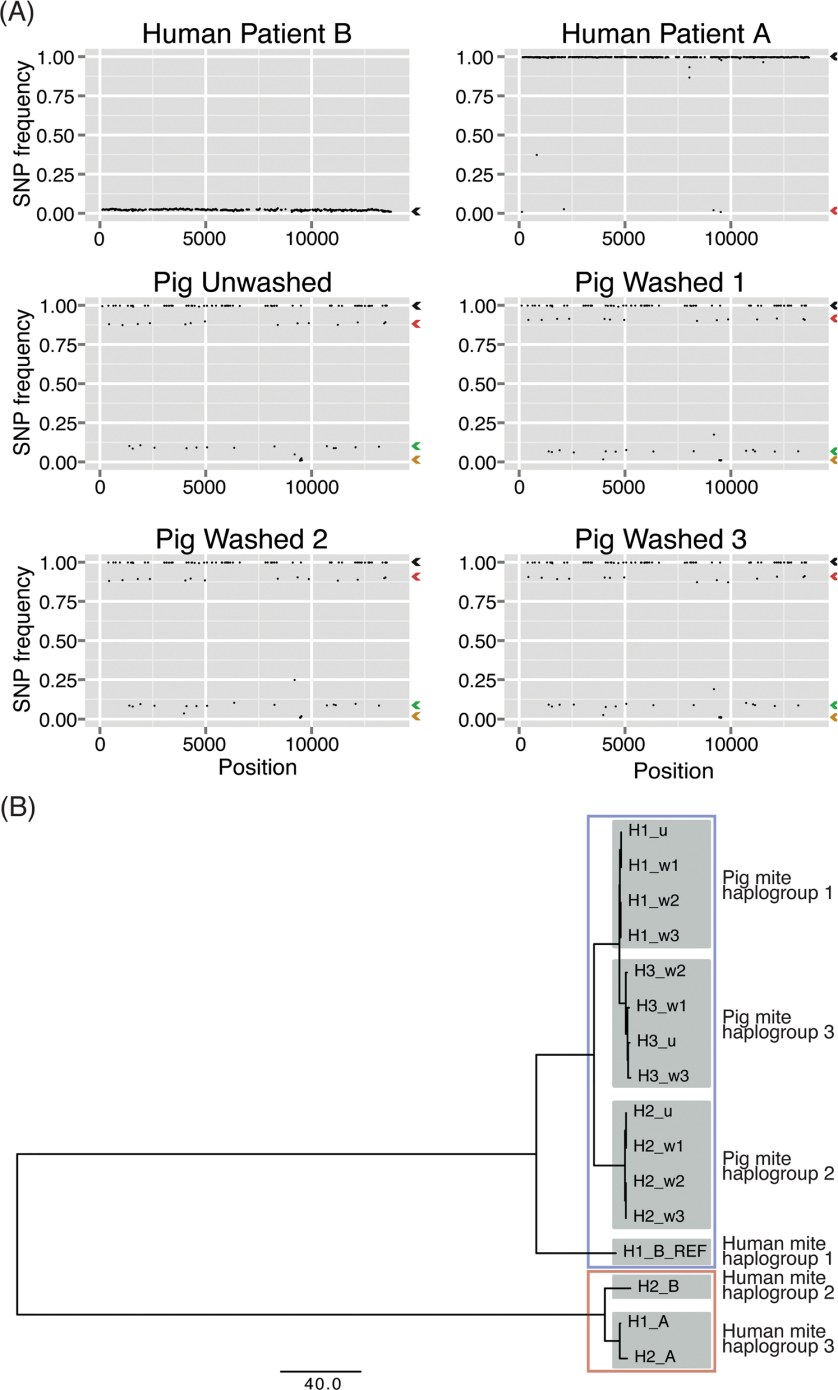
Scabies haplotype groups. (A) SNP allele frequencies in each sample show strong clustering and define haplotypes. (B) Phylogenetic tree of the inferred haplotypes from multiple human and pig samples group into 6 haplogroups and 2 broad clades (red and blue boxes).

Within each sample, SNP allele frequencies, estimated from the ratio of reads supporting the variant to the total coverage, were tightly grouped into a small number of clusters (Fig 3A), suggestive of the presence of just a few mitochondrial haplotypes in each sample. To estimate the number and frequencies of haplotypes, and to infer their sequences, we used k-means clustering (S3 Table). SNPs common to all haplotypes within a sample have a frequency of 1. Additional haplotypes are defined by the presence of extra SNPs with mean frequency less than 1. We also examined reads supporting SNPs located close to each other (<100 nt) for evidence that SNPs were on the same haplotype (supported by the same reads) or distinct haplotypes (never co-occurring on one read). Only 4 pairs of SNP were located within 100 nucleotides of each other and in two different frequency clusters. In each sample, we gave the haplotype with the highest frequency the prefix H1. Haplotypes with successively lower frequencies are labeled H2 and H3, in that order.

In the patient B sample, a single clear cluster in the SNP frequencies was observed (Fig 3A). This implies the presence of two haplotypes. The dominant haplotype, H1_B_REF, has an estimated frequency of 0.98 and corresponds to the consensus obtained from *de novo* assembly. The second haplotype (H2_B) is defined by the presence of 598 SNPs and has an estimated frequency of 0.02.

The patient A sample contains a single clear cluster, containing 593 SNPs with a frequency of 1.0. An additional 8 SNPs do not cluster well with the dominant group. These fall into four other clusters identified by k-means. We chose to ignore the three smallest clusters, resulting in just two closely-related haplotypes: H1_A, which has a frequency of 0.98, and H2_A, which has a frequency of 0.02 and is separated from H1_A by just 4 SNPs. The lack of replication of these SNPs makes the significance of these closely related haplotypes unclear.

All four pig samples contain three clear clusters, forming three haplotypes in each sample. The haplotypes with the highest frequencies (H1_u, H1_w1, H1_w2, H1_w3) have estimated frequencies of 0.89, 0.91, 0.89 and 0.90 respectively. Additional haplotypes are composed of combinations of the clusters (S3 Table) and are concordant between samples. The second haplotypes have estimated frequencies of 0.10, 0.07, 0.09 and 0.09 for the unwashed and three washed samples respectively and we label these H2_u, H2_w1, H2_w2, and H2_w3. The third haplotypes, which we label H3_u, H3_w1, H3_w2 and H3_w3 for the unwashed and three washed samples respectively, have estimated frequencies 0.02, 0.01, 0.02 and 0.01 respectively. The H1_u, H1_w1, H1_w2, H1_w3 haplotypes have 82, 82, 81, 81 SNPs; H2_u, H2_w1, H2_w2, H2_w3 have 82, 82, 81, 81 SNPs and H3_u, H3_w1, H3_w2, H3_w3 have 87, 87, 85, 87 SNPs respectively.

Similar analyses of DNA sequencing data from a limited number of individual scabies mite eggs or small pools did not identify clusters in the SNP allele frequencies (S2 Fig), suggesting that multiple haplotypes provide evidence for genetic diversity rather than heteroplasmy.

To understand the relationship between the 16 inferred haplotypes, we constructed a phylogenetic tree based on the SNPs present in each haplotype sequence using MEGA5 (v5.2.2) [51] with a distance measure based on the number of differences between haplotype sequences (S4 Table). The tree shows that the haplotypes fall into two broad clades and six haplogroups (Fig 3B). Three haplogroups are found in clinical isolates (human mite haplogroups 1–3), while the pig mites comprise three haplogroups (pig mite haplogroups 1–3). The average difference between haplotypes within each of the pig mite haplogroups is 1–2 SNPs. The two haplotypes in human mite haplogroup 3 are almost identical and very similar to human mite haplogroup 2, forming one of the clades, while human mite haplogroup 1 is distinct from the other human mite haplogroups, and more similar to the pig mite haplogroups, forming the second clade.

## Discussion

We *de novo* assembled the mitochondrial genome of the scabies mite using massively parallel sequencing data from thousands of pooled whole mites obtained from two clinical isolates from different parts of northern Australia and from a laboratory pig model. Our approach was to initially perform a full metagenomic *de novo* assembly. As expected, the samples were contaminated by bacterial reads presumably from the scabies mite gut and the scabies lesion micro-environment. We then iteratively selected Mt contigs and used these as bait to recruit and assemble genuine mite reads. Our bespoke *de novo* assembly approach has some similarity to an existing bait-and-assembly method called MITObim [52]. MITObim first directly baits the short reads using a closely related mitochondrial genome, then iteratively maps reads and performs contig extension using MIRA. De novo assembly is also supported, but only for “well behaved” data. In contrast, our approach performs full de novo assembly of the metagenomic mixture, baiting of contigs using the house dust mite genome and filtering of host mitochondrial contigs, then 1–2 iterations of read alignment and de novo assembly using velvet. Our results substantially extend the limited case studies and simulations used to validate MITObim and demonstrate that the general approach of mitochondrial genome bait-and-assembly works on real examples that are highly complex metagenomic mixtures involving both genetic heterogeneity and host/bacterial contamination.

Mitochondrial genomes are generally circular, but rare occurrences of linear mitochondrial genomes have been reported [53]. While we were unable to circularise the scabies mite mitochondrial genome assemblies, it seems unlikely that it is linear; a simpler explanation is that the region contains highly repetitive AT-rich sequences (which we observe on the flanking regions of the break) and these are difficult to map reads to and assemble across. The same region in EHDM mitochondrial genome contains the origin of replication site. This might also be a factor for the missing coverage in this region.

**Fig 4:**
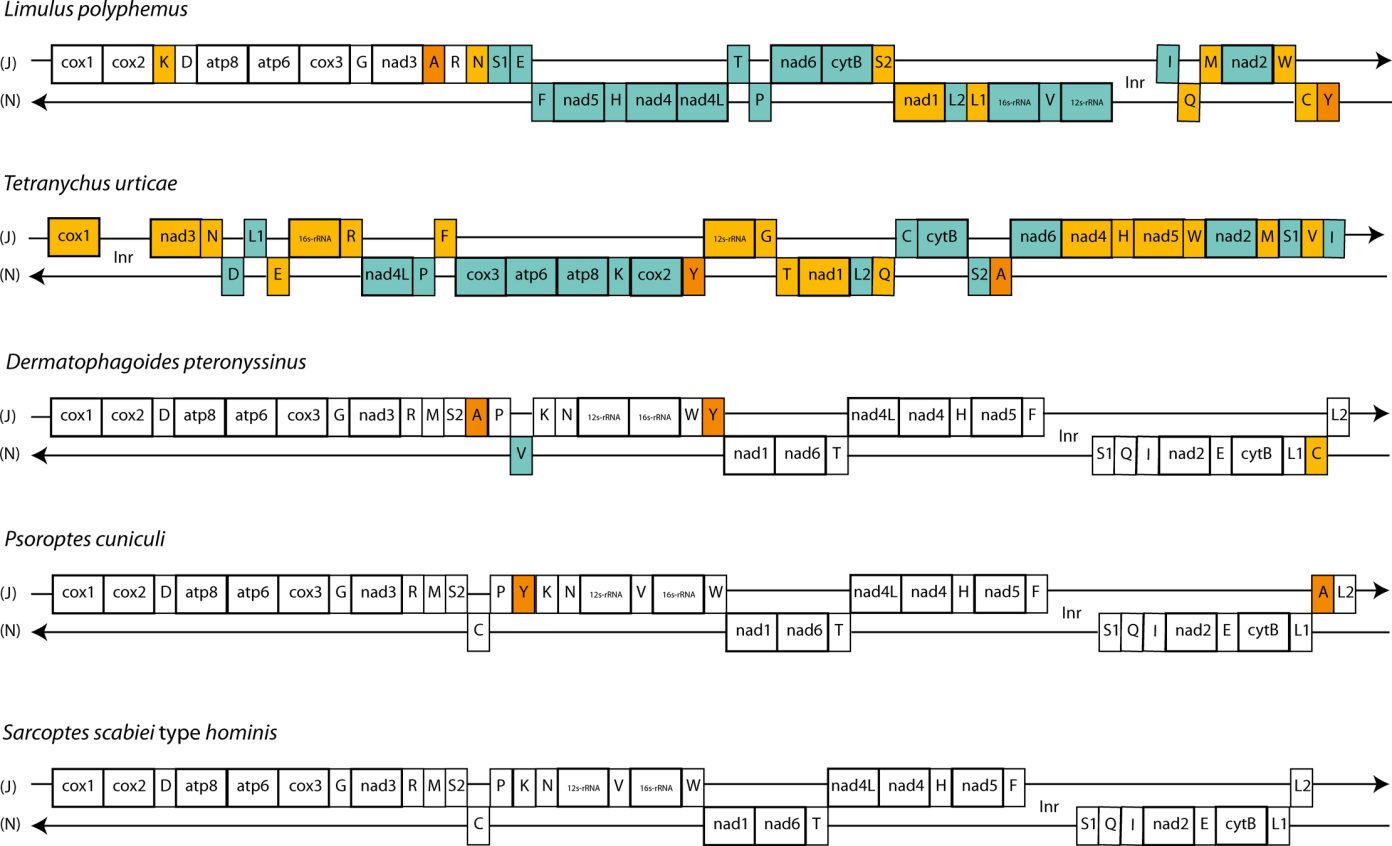
Mitochondrial gene arrangements of selected Acari species and *Limulus polyphemus*, representing the arthropod ground pattern. Genes are drawn according to order, intergenic distances are not shown and genes sizes are not drawn according to scale. J stands for majority strand and N stands for minority strand. *L*. *polyphemus* is included in the comparison representing the arthropod ground pattern of gene arrangement for chelicerates. Yellow boxes represent genes that have different positions relative to scabies mite; teal boxes represent genes that are different in terms of both position and orientation; and orange boxes represent genes that are not present in scabies mite. Accession numbers of the Mt genomes are listed in S5 Table.

The gene content and organisation of the *Sarcoptes scabiei* var. *hominis* mitochondrial genome is the same as the recently published var. *canis* mitochondrial genome contig (NCBI GenBank: CM003133 JXLN01000000) [54]. The gene order is also similar to the rabbit ear mite, *Psoroptes cuniculi*, mitochondrial genome (NCBI Refseq: NC_024675) [55] with the exception of the two additional tRNA genes found in the latter (Fig 4) and the house dust mite (NCBI GenBank: EU884425), suggesting a close evolutionary relationship between these species. These observations further support the hypothesized close evolutionary relationship between the parasitic scabies mite and free-living house dust mite [56, 57]. However, it is distinct from most other Acari mitochondrial genomes sequenced to date [40], including *Tetranychus urticae* (NCBI GenBank: EU345430). It is also quite distinct from *Limulus polyphemus* (NCBI GenBank: NC_003057), which represents the arthropod ground pattern of gene arrangement for chelicerates [58].

We found variations in the diversity of infestations between individuals (clonal or heterogeneous). Mites from skin scrapings from one patient with crusted scabies (patient A) appeared to be essentially clonal. This lack of diversity suggests that either a single female mite may initiate infection, or selection by the host immune system may operate. Mites from the second patient (patient B), who had more severe crusted scabies than patient A, showed two highly divergent haplotypes. Remarkably, one of the haplotypes in the patient B sample was much more similar to the pig mite haplotypes than to the other human mite haplotypes. This may reflect an actual cross-species infection, highlight the possibility of this, or could merely suggest that the genetic differences between varieties are limited.

The population-level genetic diversity of scabies mites has been studied extensively using single or small numbers of gene sequences. One recent study based on cox1 gene sequences showed that scabies infestations of humans, dogs and other animals fall into 3 clades [21]. Two clades contained only geographically isolated var. *hominis* mites, while the third contained closely related human and animal mites. Another recent study, which used 3 gene sequences, reported 5 clades—4 distinct var. *hominis* clades (one for each geographical region studied) and one clade containing closely-related var. *hominis* and other animal scabies mite varieties [21, 22]. These findings are consistent with our observation that one of the patient B mite haplotypes was more similar to the pig mite haplotypes than to the other human mite haplotypes. Since we used the entire mitochondrial genome, we expect our results to be robust. Moreover, our intra-sample inference of haplotypes has revealed that patients and animals can harbour multiple genetically divergent mites, or near clonal infestations. To the best of our knowledge, this is the first view of intra-host genetic diversity for scabies mites.

One issue with our analysis of haplotypes is that we are unable to utilize a few unclustered and unreplicated SNPs, in particular, from the patient B sample. These may define additional haplotypes or represent genetic drift. Notwithstanding, these haplotypes would be extremely closely related. Our justification is that these are very small differences between haplotypes and may be due to sequencing errors and thus erroneously detected variants, whereas large numbers of SNPs with clustered frequency are more likely to be real. Regardless, our analysis provides a first look at the genetic diversity of scabies infections within patients. However, further study with broader population sample is required to refine the intra- and inter-host population diversity structure.

### Conclusion

Scabies is responsible for significant morbidity in Indigenous Australians in many remote communities of northern and central Australia. Effective control and prevention of scabies epidemics in those communities is of paramount importance as scabies has a long-term effect on the life expectancy and quality. We have sequenced, assembled and annotated the mitochondrial genome of the scabies mite using a bespoke bait-and-assembly approach; we identified SNPs in multiple isolates from patients and a laboratory pig model, and inferred the haplotype structure and diversity of individual infections. We used these tools to investigate the genetic diversity within individual infestations. Larger samples are now needed.

The development of genomics resources for studying the scabies mite will accelerate research into this parasite, just as genome sequences have for other neglected parasitic diseases. For example, in malaria genomic resources provided means to identify drug resistance causing mutations [59] or in schistosomiasis it helped suggesting new approaches to preventions and strategies for control [60]. The scabies mite mitochondrial genome sequence will facilitate further population genetics research in this area.

## Supporting Information

S1 Fig Metagenomic profile of the patient B sample. Plots generated using PhymmBL [37] and Krona [61]. (A) The patient B assembly has nearly half of the contigs belonging to contaminants. (B) Realigning reads back to contigs shows species abundance in the patient B sample reads used in the assembly.
(TIF)

S2 Fig SNP allele frequencies in sequence data from single and small pools of scabies mite eggs (*S*. *scabiei* var. *suis*). Short read sequencing data of DNA from single eggs, or pools of 5 or 16 eggs were aligned to the scabies mite Mt reference genome (patient B), and SNPs called using the method described previously (except the minimum coverage threshold was set to 15 in this case due to lower coverage). The low frequency SNPs in the pooled egg samples (5 and 16) are all adjacent to homopolymer runs and consistent to be sequencing errors. The cluster of SNPs with frequency 1.0 and absence of other clear clusters suggests that only a single haplotype is present in individual eggs and that genetic diversity, rather than heteroplasmy explains the clusters in other samples.
(TIF)

S1 Table Details of sequencing libraries. (XLSX)

S2 Table Single nucleotide polymorphisms called by Varscan on all samples along with their corresponding base positions. (TXT)

S3 Table Results from k-means clustering showing SNP frequencies and formation of haplotypes based on frequency clustering. (XLSX)

S4 Table Pairwise distance between haplotypes in a matrix format. Here, distance is represented by number of base differences between haplotype sequences.
(XLS)

S5 Table Accession numbers of Mt genomes used for gene arrangement comparison. (XLSX)
