# Supplementary figures and images for: Mitochondrial Genome Sequence of the Scabies Mite Provides Insight into the Genetic Diversity of Individual Scabies Infections
Source: PLoS Negl Trop Dis. 2016 Feb 12;10(2):e0004384. doi: 10.1371/journal.pntd.0004384 (PMC4752359; doi:10.1371/journal.pntd.0004384)

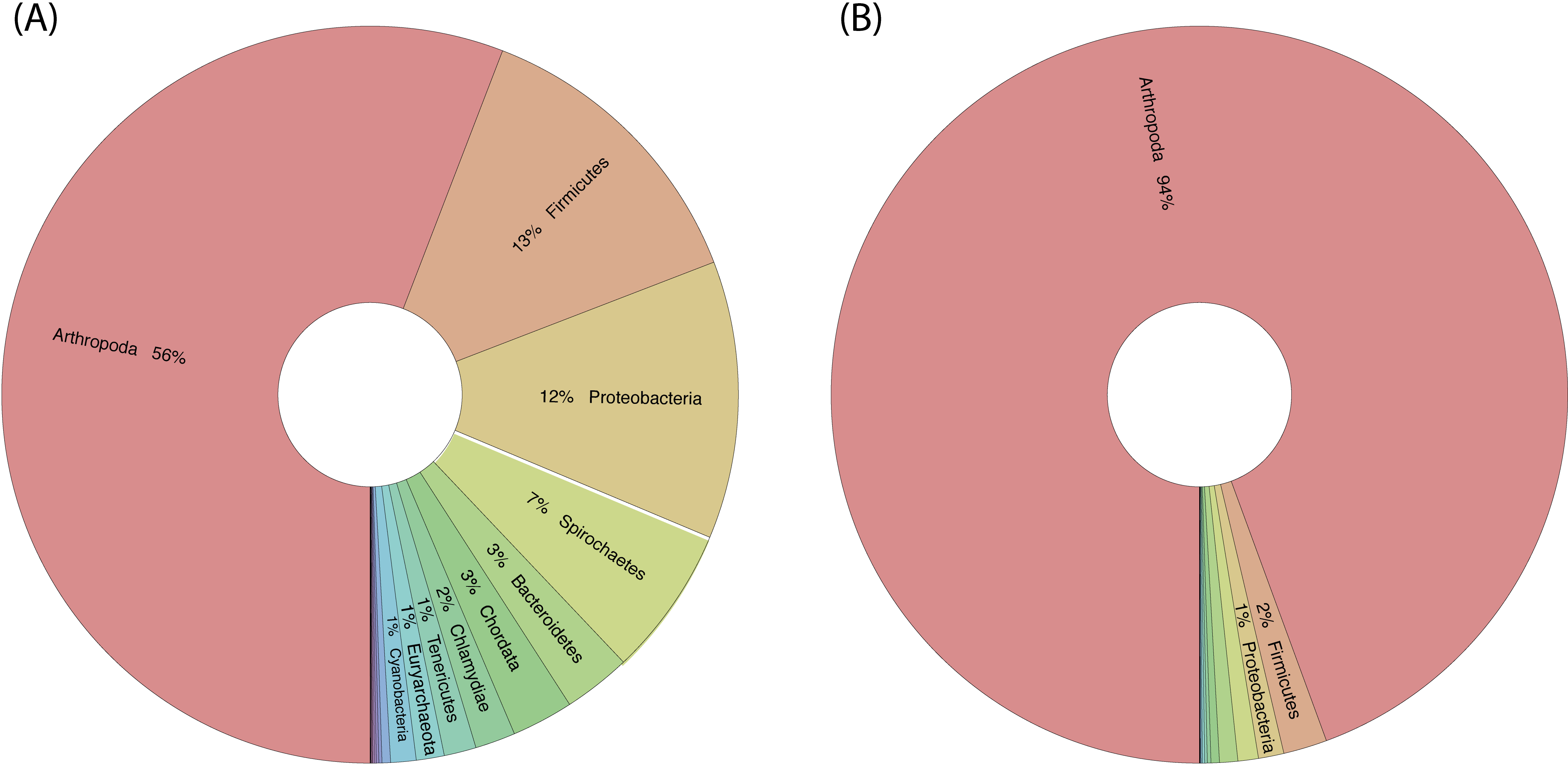

Supplement: S1 Fig — Plots generated using PhymmBL [37] and Krona [61]. (A) The patient B assembly has nearly half of the contigs belonging to contaminants. (B) Realigning reads back to contigs shows species abundance in the patient B sample reads used in the assembly. (TIF) [file pntd.0004384.s001.tif]

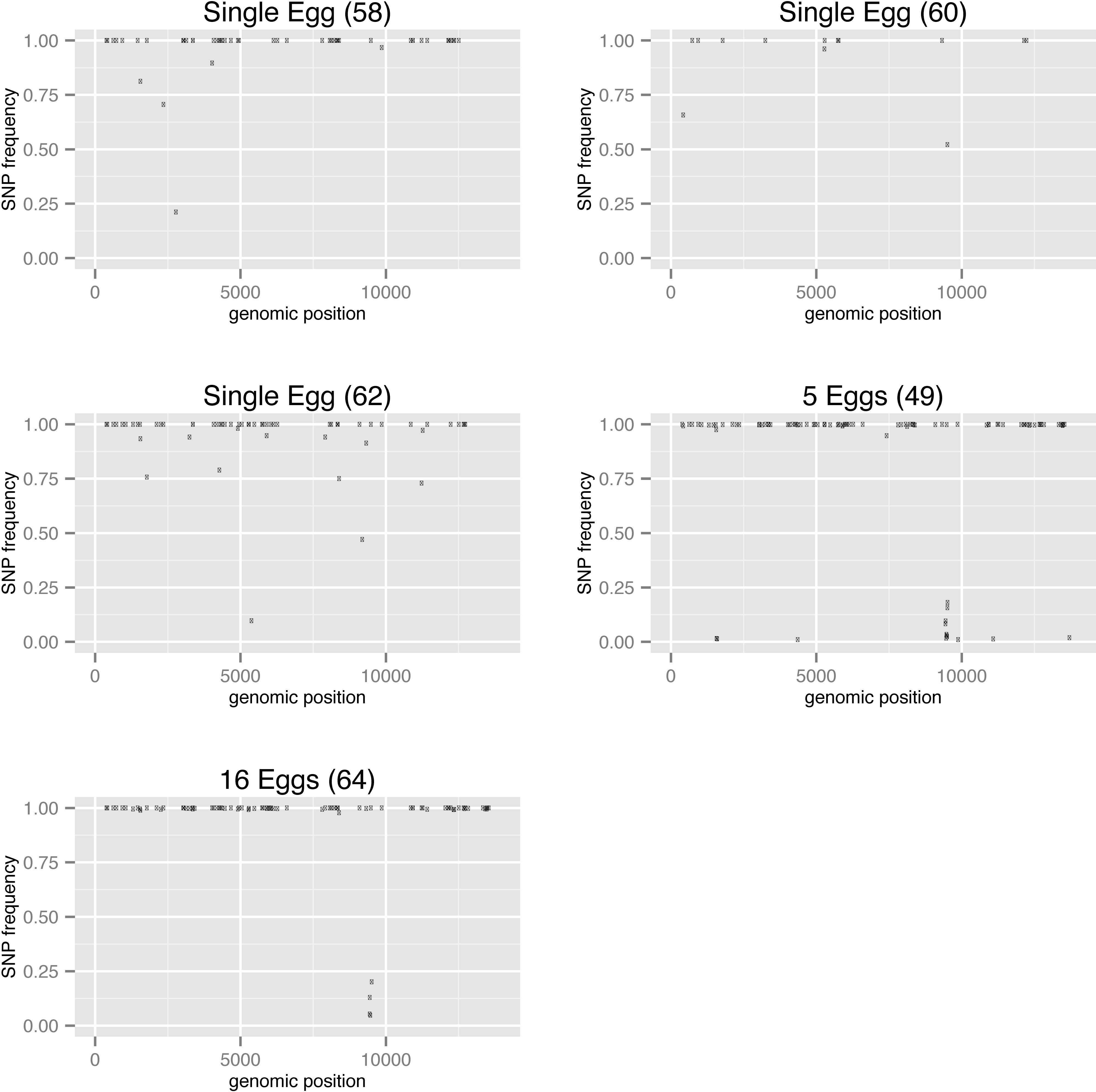

Supplement: S2 Fig — Short read sequencing data of DNA from single eggs, or pools of 5 or 16 eggs were aligned to the scabies mite Mt reference genome (patient B), and SNPs called using the method described previously (except the minimum coverage threshold was set to 15 in this case due to lower coverage). The low frequency SNPs in the pooled egg samples (5 and 16) are all adjacent to homopolymer runs and consistent to be sequencing errors. The cluster of SNPs with frequency 1.0 and absence of other clear clusters suggests that only a single haplotype is present in individual eggs and that genetic diversity, rather than heteroplasmy explains the clusters in other samples. (TIF) [file pntd.0004384.s002.tif]
